# Coronary Artery Fistula Causing Acute Myocardial Infarction and Right Ventricle Thrombus

**DOI:** 10.7759/cureus.2314

**Published:** 2018-03-13

**Authors:** Eduardo L Santos, Milena M Gouveia, Ricardo F Silva, Renata Ávila, Maria A Aquino, Luca T Dompieri, Renato D Lopes

**Affiliations:** 1 Department of Cardiology, UFPE; 2 Cardiology Unit, Dom Helder Camara Hospital; 3 Cardiology Unit, Esperança Hospital; 4 Medical School, Federal University of Pernambuco; 5 Division of Cardiology, Duke University Medical Center

**Keywords:** coronary artery fistulae, aneurism, acute myocardium infarction, thrombus in right ventricle, coronary fistula, coronary artery fistula

## Abstract

Coronary artery fistula (CAF) is a rare congenital anomaly, which is most commonly asymptomatic. In symptomatic cases, aneurysms can occur with complications of thromboembolic events. This report describes a rare case of CAF presenting with complications of inferior acute myocardial infarction and thrombus formation in the right ventricle.

## Introduction

Coronary artery fistula (CAF) is a rare congenital anomaly which is asymptomatic in most cases. Potential complications include aneurysm with spontaneous rupture, myocardial ischemia, thrombosis, and thromboembolic events [1]. We describe a case of CAF causing acute myocardium infarction and formation of thrombus in the right ventricle.

## Case presentation

**Figure 1:**
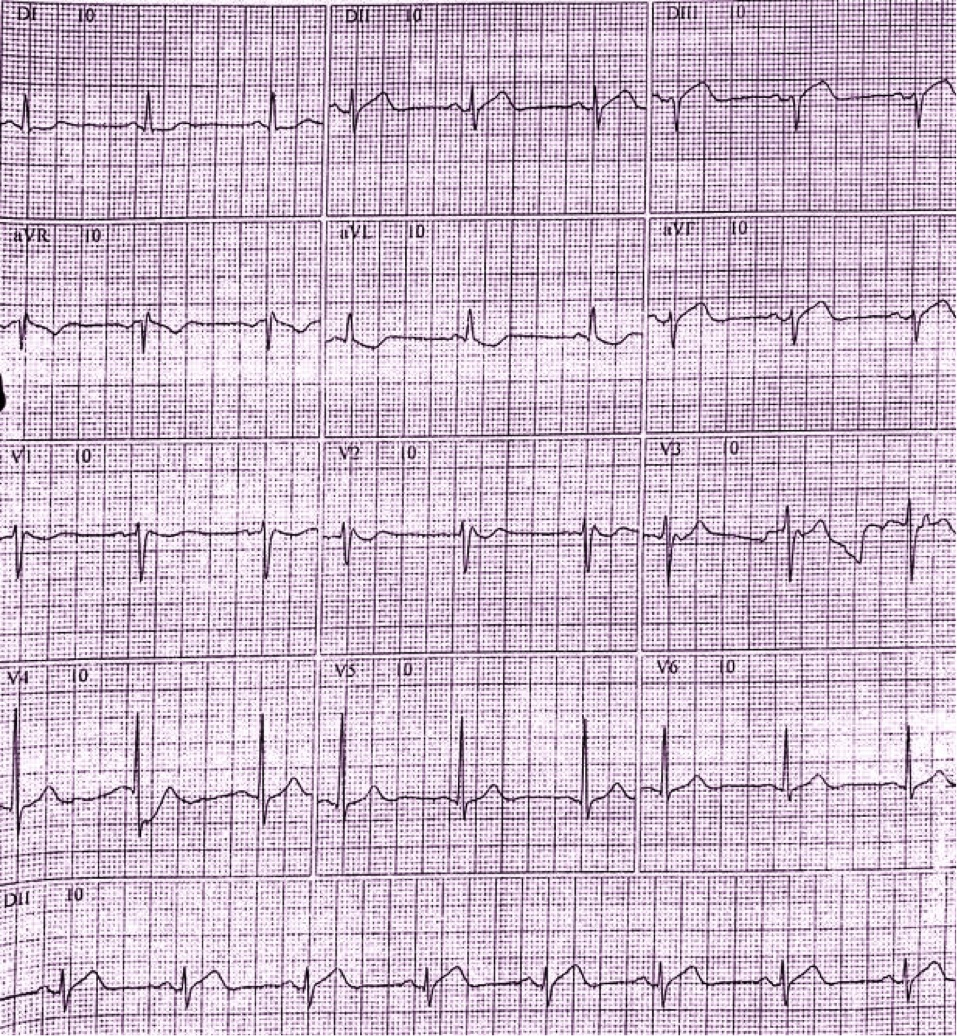
Electrocardiogram (ECG) ECG with slight ST-segment elevation in leads DIII and aVF. Reciprocal changes are seen in DI and aVL.

A 40-year-old male was admitted to the primary care unit with typical chest pain and dyspnea for 14 hours. The admission electrocardiogram (ECG) demonstrated a slight ST-segment elevation in DIII and aVF associated with ST-segment depression in DI and aVL. An inverted T wave was seen in the latter (Figure 1). 

**Figure 2:**
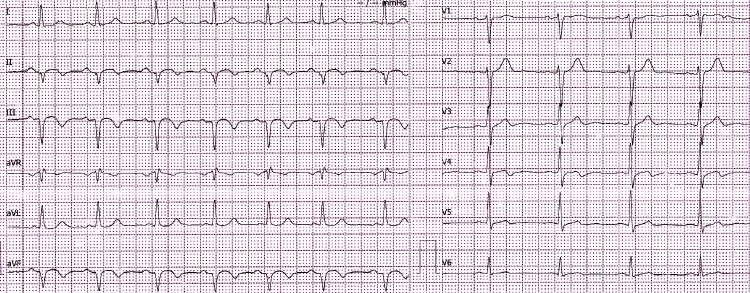
Electrocardiogram (ECG) at PCI-capable hospital showed pathological Q waves in inferior leads in the absence of ST segment elevation PCI: percutaneous coronary intervention.

Clopidogrel and aspirin were administered before the patient was transferred to a percutaneous coronary intervention (PCI)-capable hospital. At admission, the ECG already showed pathological Q waves in inferior leads (Figure 2). 

Coronary angiography was performed and showed right coronary aneurysm and occlusion. Since the patient had more than 12 hours of the initial presentation and was asymptomatic at the time of the exam, PCI was not performed.

**Figure 3:**
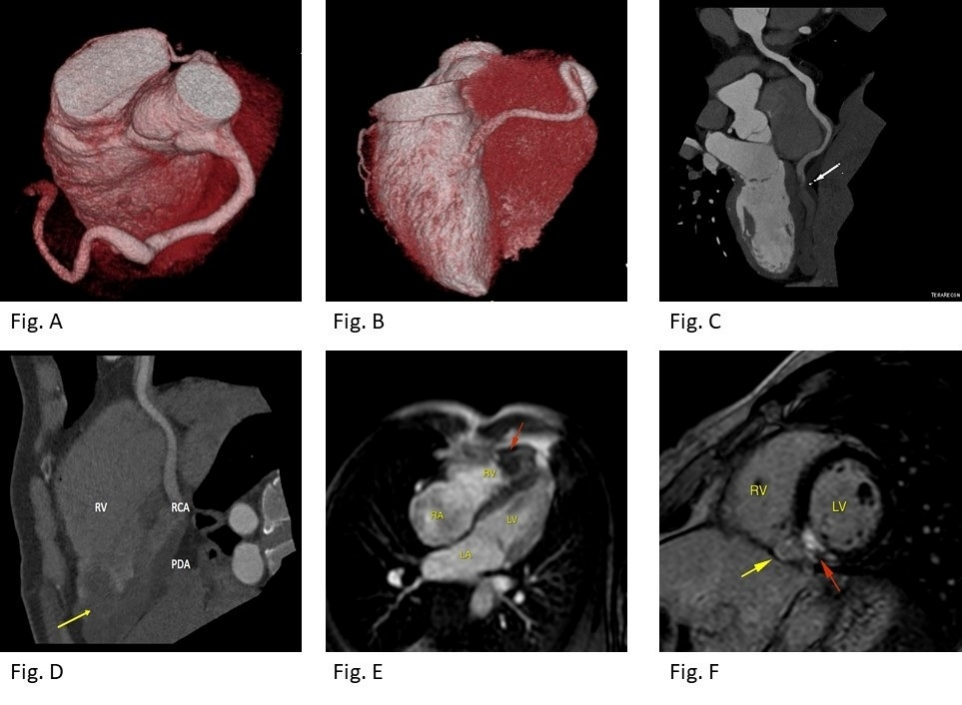
Cardiac computed tomography (CCT) A and B: CCT three-dimensional reconstruction (volume rendering technique) demonstrates an aneurysm in right coronary artery (RCA) with total oclusion in the distal segment. C and D: Curved multiplanar reformation (MPR) shows thrombus in distal RCA and posterior descending artery (PDA) (white arrow) with extension to the right ventricle (RV) (yellow arrow). E. Long-axis of first-pass myocardial perfusion on cardiac magnetic resonance imaging (MRI) demonstrates a large thrombus image inside the RV. F. Left ventricle (LV) late gadolinium enhancement short-axis on cardiac MRI. Note delayed enhancement in the LV inferoseptal segment of basal region (red arrow) and right ventricle involvement (yellow arrow) compatible with myocardial infarction. RA: right atrium; LA: left atrium.

Cardiac computed tomography (CCT) confirmed an aneurysm in the right coronary artery (RCA) and its branches. It revealed a thrombus in the distal RCA and posterior descending and posterior ventricular branches with extension to the right ventricle’s apex due to CAF between the posterior descending artery and the right ventricle (Figures 3A-3D). Cardiac magnetic resonance (CMR) was performed and demonstrated vascular saccular aneurysm dilatation in the right ventricle anteroinferior surface with a large thrombus image inside, measuring 3.5 x 3.4 x 2.4 cm and communicating with the right ventricle apical region (Figure 3E). A late myocardial enhancement was observed in the left ventricle inferoseptal segment of the basal region and right ventricle involvement compatible with myocardial infarction (Figure 3F).

The patient is now asymptomatic under ambulatory follow-up. 

## Discussion

CAF is an abnormal communication between a coronary artery and a cardiac chamber or other vessels [2]. It is often due to congenital anomalies but can also be acquired from trauma or from invasive procedures such as endomyocardial biopsy [1]. The exact etiology of congenital cases is unknown; however, the failure of obliteration of the intramyocardial trabecular sinusoids, with anomalous development of the intra-trabecular spaces, is the possible cause [2].

CAF commonly arise from the right coronary artery (55%) followed by the left anterior descending artery (35%). Over 90% of fistulas flow in the direction of right-heart structures [2]. Drainage into the left ventricle is less frequent (3 % of CAFs) [3].

The majority of patients are asymptomatic [4]. However, medium to large sizes CAF can present with clinical manifestations such as angina pectoris, myocardial infarction, progressive dilatation, heart failure, pulmonary hypertension, thrombosis of the fistula, and formation of aneurysms with possible ruptures [1, 3-4]. 

Aneurysms can occur in up to 19% of cases [2]. The main complications include spontaneous rupture, myocardial ischemia, thrombosis, and thromboembolic events [1-3].

CMR imaging and CCT are useful, noninvasive, and accurate imaging techniques for the detection of major coronary artery anomalies. In the presented case, the distal part of the posterior descending artery developed thrombosis and a fistula draining into the right ventricle. It leads to thrombus formation in the right ventricle and acute myocardium infarction.

## Conclusions

CAF is a rare congenital anomaly which is most commonly asymptomatic. We describe a rare case of symptomatic CAF complicating with acute myocardium infarction and formation of thrombus in the right ventricle.
